# Impact of roasting on the phenolic and volatile compounds in coffee beans

**DOI:** 10.1002/fsn3.2849

**Published:** 2022-04-01

**Authors:** Hanjing Wu, Peiyao Lu, Ziyao Liu, Javad Sharifi‐Rad, Hafiz A. R. Suleria

**Affiliations:** ^1^ School of Agriculture and Food Faculty of Veterinary and Agricultural Sciences The University of Melbourne Parkville Victoria Australia; ^2^ Facultad de Medicina Universidad del Azuay Cuenca Ecuador

**Keywords:** antioxidant properties, characterization, *Coffea arabica*, GC‐MS, LC‐MS/MS, phenolic compounds, roasting, semi‐quantification, volatile compounds

## Abstract

Phenolic compounds present in coffee beans could generate flavor and bring benefits to health. This study aimed to evaluate the impacts of commercial roasting levels (light, medium, and dark) on phenolic content and antioxidant potential of Arabica coffee beans (*Coffea arabica*) comprehensively via antioxidant assays. The phenolic compounds in roasted samples were characterized via liquid chromatography–electrospray ionization quadrupole time‐of‐flight mass spectrometry (LC‐ESI‐QTOF‐MS/MS). Furthermore, the coffee volatile compounds were identified and semi‐quantified by headspace/gas chromatography–mass spectrometry (HS‐SPME‐GC‐MS). Generally, for phenolic and antioxidant potential estimation, light roasted samples exhibited the highest TPC (free: 23.97 ± 0.60 mg GAE/g; bound: 19.32 ± 1.29 mg GAE/g), DPPH, and FRAP. The medium roasted beans performed the second high in all assays but the highest ABTS^+^ radicals scavenging capacity (free: 102.37 ± 8.10 mg TE/g; bound: 69.51 ± 4.20 mg TE/g). Totally, 23 phenolic compounds were tentatively characterized through LC‐ESI‐QTOF‐MS/MS, which is mainly adopted by 15 phenolic acid and 5 other polyphenols. The majority of phenolic compounds were detected in the medium roasted samples, followed by the light. Regarding GC‐MS, a total of 20 volatile compounds were identified and semi‐quantified which exhibited the highest in the dark followed by the medium. Overall, this study confirmed that phenolic compounds in coffee beans would be reduced with intensive roasting, whereas their antioxidant capacity could be maintained or improved. Commercial medium roasted coffee beans exhibit relatively better nutritional value and organoleptic properties. Our results could narrow down previous conflicts and be practical evidence for coffee manufacturing in food industries.

## Graphical abstract

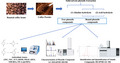

## 1 INTRODUCTION

Coffee is gradually becoming one of the main commercial food products and world's most widely consumed beverages (Farah, 2012; Valduga et al., 2019). *Coffea arabica* (Arabica) is one of the major commercial cultivars which takes about 70% of global coffee markets (Rajesh Banu et al., 2020; Waters et al., 2017). Generally, bioactive compounds in coffee could be divided into three major categories, phenolic compounds, flavonoids, and alkaloids. Based on the antioxidant and anti‐inflammatory properties of these present bioactive compounds, numerous researchers pointed that regular drinking of coffee could reduce the risk of some chronic diseases including type II diabetes, cardiovascular and autoimmune diseases, and certain types of cancer (Harumi Kondo & Ikewaki, 2012).

Phenolic compounds are second metabolites that commonly exist in higher plants and beverages of plant origin (Farah & Donangelo, 2006). It is present mostly as free (soluble) in plant cell vacuoles and bound (insoluble) forms bound to the cell wall polymeric molecules by ester and glycoside bonds. Hence, the extraction of bound phenolic compounds should use alkaline or acid hydrolysis rather than aqueous organic solvents directly (Mehari et al., 2020). Lu et al. (2020) indicated that the predominant phenolic compounds present in coffee beans are chlorogenic acids (CGAs), particularly 5‐*O*‐caffeoylquinic acid (5‐CQA). Chlorogenic acids refer to a class of esters derived from several certain hydroxycinnamic acids and quinic acid, including *p*‐coumaroylquinic, feruloylquinic, and caffeoylquinic acids (Gokcen & Sanlier, 2019). Commonly, the content of chlorogenic acids in Arabica ranges from 4% to 8.4%, meanwhile the concentration is increasing with the maturity of coffee beans (Stalmach, 2012). Chlorogenic acids contribute to the bitter, acid, and astringent flavor of coffee brew, especially because of caffeoylquinic and feruloylquinic acids (Farah, 2012). It also could improve the nutritional value of coffee brew due to its high antioxidant, antibacterial, antiviral, and chemo‐preventive capacity (Gokcen & Sanlier, 2019). However, the decomposition of CGAs could easily occur if the temperature is higher than 80°C during processing owing to the thermal instability of polyphenols (Król et al., 2020). Therefore, the physicochemical characteristics of coffee beans are considerably determined by processing conditions (Cordoba et al., 2020; Farah et al., 2005, 2006).

Roasting is the most essential procedure during the processing chain to generate aroma and flavor (Baggenstoss et al., 2008). Aroma is an important attribute for the acceptance of coffee beans. Different varieties of coffee beans, their natural origin and processing, especially roasting, will contribute to a variable volatile composition (Somporn et al., 2011). Commercially, the temperature required for three coffee roasting degrees (light, medium, and dark) should be between 195°C and 245°C (Somporn et al., 2011). In chemical aspects, Maillard reaction, nonenzymatic reaction, browning reaction, and Strecker degradation would take place (Farah, 2012; Somporn et al., 2011). The composition of volatiles in green coffee beans, such as aldehydes, ketones, furans, acetic, propanoic, butanoic acid, and other compounds, could be changed during roasting due to those reactions (Somporn et al., 2011). Meanwhile, the interactions would result in the changes in composition involving the loss of polysaccharides, oligosaccharides, chlorogenic acids, and trigonelline, and the generation of lactones of the chlorogenic acids so that influence the antioxidant activities of coffee beans eventually (Baggenstoss et al., 2008). Additionally, melanoidins would be generated via Maillard reaction between amino acids and reducing sugars (Farah & Donangelo, 2006). Quinic acid would be generated as well (Perez‐Burillo et al., 2019). Melanoidins and quinic acid are considered as bioactive compounds which could improve the antioxidant, antibacterial, and metal chelating properties of coffee beans (Farah, 2012). Therefore, the total antioxidant activities of coffee beans would be partially maintained.

Therefore, this research aimed to assess the impact of commercial roasting degrees (light, medium, and dark) on the content and the composition of phenolic and volatile compounds of coffee beans as well as their antioxidant potential. Total phenolic (TPC), flavonoids (TFC), and condensed tannins (TCT) content, reducing powder (RPA), ferric reducing antioxidant potential (FRAP), and ferrous ion chelating activity (FICA), 2,2’‐diphenyl‐1‐picrylhydrazyl (DPPH), 2,2’‐azinobis‐(3‐ethylbenzothiazoline‐6‐sulfonic acid) (ABTS) and hydroxyl radical scavenging activity (^·^OH‐RSA) were applied to estimate the antioxidant potential of phenolic compounds with the combination of ultraviolet‐visible spectroscopy. Liquid chromatography–electrospray ionization quadrupole time‐of‐flight mass spectrometry (LC‐ESI‐QTOF‐MS/MS) was used for the characterization and identification of phenolic compounds. Furthermore, headspace/gas chromatography–mass spectrometry (HS‐SPME‐GC‐MS) was applied for the identification and quantification of volatile compounds in the roasted coffee beans.

## 2 MATERIALS AND METHODS

### 2.1 Chemical and reagents

Water for all assays was Milli‐Q water (deionized) obtained via Millipore Milli‐Q Gradient Water Purification System (Darmstadt, Germany). The standards for antioxidant assays included gallic acid, quercetin, catechin, L‐ascorbic acid, and 6‐hydroxy‐2,5,7,8‐tetramethylchroman‐2‐carboxylic acid (Trolox) were obtained from Sigma‐Aldrich (St. Louis, MO, USA).

For the estimation of polyphenols and antioxidant potential, sodium carbonate anhydrous, sodium hydroxide pellets, and hydrogen peroxide (30%) were purchased from Chem‐Supply Pty Ltd. (Adelaide, SA, Australia) and 98% sulfuric acid was obtained from RCI Labscan Ltd. (Bangkok, Thailand). The rest of all the chemicals were purchased from Sigma‐Aldrich (Castle Hill, NSW, Australia), including Folin–Ciocalteu reagent, hydrated sodium acetate, hexahydrate aluminum chloride, vanillin, 2,2’‐diphenyl‐1‐picrylhydrazyl (DPPH), 2,4,6‐tripyridyl‐s‐triazine (TPTZ), 2,2’‐azino‐bis(3‐ethylbenzothiazoline‐6‐sulfonic acid) (ABTS), potassium persulfate, ferric chloride (Fe [III]Cl_3_·6H_2_O), ferric (III) chloride anhydrous, sodium phosphate dibasic heptahydrate, sodium phosphate monobasic monohydrate, iron (II) chloride, iron (II) sulfate heptahydrate, 3‐hydroxybenzoic acid, ferrozine, and potassium ferricyanide.

### 2.2 Sample preparation

Roasted coffee beans samples with light‐, medium‐, and dark‐roasted levels used in the intended research project were purchased from Seven Seeds Company, a local coffee retail in Melbourne, Australia. Roasted coffee beans were milled into dried powder with a mean particle size by coffee grinder (Russell Hobbs Classic, model DZ‐1613, Melbourne, VIC, Australia) and then stored at −20°C in dark area before extraction.

### 2.3 Extraction of free and bound phenolic compounds

The extraction of free phenolic compounds from coffee samples was performed as per the methods described by Peng et al. (2019) with some modifications. Coffee powder was fully mixed with 70% ethanol at 1:10 (w:w) and homogenized for 30 s at 10,000 rpm by Ultra‐Turrax T25 Homogenizer (IKA, Staufen, Germany) followed by 12 h incubation under 4°C at 120 rpm in a shaking incubator (ZWYR‐240 incubator shaker, Labwit, Ashwood, VIC, Australia). Mixture was then centrifuged for 15 min at 5000 rpm under 4°C using Hettich Refrigerated Centrifuge (ROTINA380R, Tuttlingen, BadenWürttemberg, Germany). The supernatant fluid was filtered via 0.45 µm syringe filter (Thermo Fisher Scientific Inc., Waltham, MA, USA) and collected as free phenolic extracts.

Bound phenolic compounds in samples were extracted based on Phan et al. (2019) method with some modifications. The sediment went through alkaline hydrolysis by adding 2 M NaOH and incubating for 1 h at 200 rpm in the shaking incubator. Afterwards, concentrated HCl was added to adjust pH 2.0 for acid hydrolysis and recovered pH to 7.0 with 2 M NaOH. Then, the samples were mixed with 70% ethanol and incubated for 60 min to dissolve the released bound phenolic compounds into the organic solvent phase. The mixture was centrifuged for 20 min at 8000 rpm under 4°C. The supernatant fluid was collected and filtered by syringe filter as bound phenolic extracts. Both free and bound phenolic extracts were stored under −20°C and ready for further analysis.

### 2.4 Quantification of phenolic compounds and antioxidant assays

All estimated analyses for phenolic compounds (TPC, TFC, and TCT), as well as the determination of total antioxidant capacity (DPPH, ABTS, FRAP, ^·^OH‐RSA, FICA, and RPA), were modified to adapt to the 96‐well plate (Costar, Corning, NY, USA) and spectrophotometer (Multiskan^®^ Go microplate photometer) (Thermo Fisher Scientific, Waltham, MA, USA) according to Suleria et al. (2020) and Ali et al. (2021) with modifications.

#### 2.4.1 Determination of total phenolic content (TPC)

The total content of phenolic compounds in coffee beans was estimated through Folin–Ciocalteu method with some modifications based on Mussatto et al. (2011). Briefly, 25 μl sample extract or standard, 25 μl Folin–Ciocalteu reagent solution and 200 μl water were added into plate followed by 5 min incubation at 25°C. Subsequently, 25 μl of 10% (w/w) sodium carbonate was added followed by 1 h incubation under the same conditions. Gallic acid (0–200 μg/ml) and water were used as calibration curve and blank, respectively. Absorbance was measured at 765 nm and the results were expressed as mg gallic acid equivalents (GAE) per gram based on dry weight (mg GAE/g) ± standard deviation (SD).

#### 2.4.2 Determination of total flavonoid compounds (TFC)

The total flavonoids content of roasted coffee beans was determined according to Ali et al. (2021). Briefly, 80 μl sample extract, 80 μl 2% aluminum chloride, and 120 µl 50 g/L sodium acetate solution were added into plate in sequence, followed by 2.5 h incubation in the dark at 25°C. Quercetin (0–50 μg/ml) and water were used for standard curve and blank, respectively. Absorbance was measured at 440 nm and the final content of flavonoids in samples was expressed as mg quercetin equivalents (QE) per dry weight (mg QE/g) ± SD.

#### 2.4.3 Determination of total condensed tannins (TCT)

The total content of condensed tannins in coffee beans was quantified through the vanillin sulfuric acid method according to Ali et al. (2021). Briefly, 25 μl sample extract, 150 μl vanillin solution, and 25 μl 32% sulfuric acid were injected into plate and incubated for 15 min in the dark under 25°C. Catechin (0–1 mg/ml) and water were used for standard curve and blank, respectively. The absorbance was measured at 500 nm and converted into the final content of condensed tannins in coffee beans as mg catechin equivalents (CE) per dry weight (mg CE/g) ± SD.

#### 2.4.4 2,2’‐diphenyl‐2‐picryl‐hydrazyl (DPPH) antioxidant assay

Modified DPPH assay based on the method of Nebesny and Budryn (2003) was used as a preliminary test for the evaluation of free radical scavenging activity of coffee beans with the change in color from purplish to yellowish. Briefly, 40 μl sample extract or standard and 260 μl 0.1 mM DPPH solution were added into plate and incubated for 30 min at 25°C. Trolox (0–200 μg/ml) and water were used for standard curve and blank, respectively. The absorbance was measured at 517 nm and the results were expressed as mg Trolox equivalents (TE) per dry weight (mg TE/g) ± SD.

#### 2.4.5 2,2’‐azinobis‐(3‐ethylbenzothiazoline‐6‐sulfonic acid) (ABTS) assay

Modified ABTS^+^ decolorization assay according to the method of Re et al. (1999) was also conducted to evaluate coffee beans antioxidant capacity. ABTS^+^ dye solution was prepared by mixing 5 ml 7 mM ABTS^+^ solution and 88 μl 140 mM potassium persulfate followed by 16 h incubation in the darkroom. Subsequently, 10 μl sample extract and 290 μl dye solution were added into plate and incubated for 6 min at 25°C. Trolox (0–500 μg/ml) and water were used for calibration curve and blank, respectively. The absorbance was measured at 734 nm and the results were expressed as mg TE/g ± SD.

#### 2.4.6 Ferric reducing antioxidant power (FRAP) assay

FRAP assay was conducted according to the method of Benzie and Strain (1996) with modifications. The FRAP dye solution was prepared in the dark by mixing 300 mM sodium acetate solution, 10 mM TPTZ solution, and 20 mM Fe [III] solution at a ratio of 10:1:1 (v/v/v). Briefly, 20 μl sample extract and 280 μl dye solution were added into plate and incubated for 10 min at 37°C. Trolox (0–200 μg/ml) and water were used for calibration curve and blank, respectively. The absorbance was measured at 593 nm and the results were expressed as mg TE/g ± SD.

#### 2.4.7 Estimation of hydroxyl radical scavenging activity (^·^OH‐RSA)

Modified Fenton‐type reaction method according to the method of Smirnoff and Cumbes (1989) was used for the evaluation of hydroxyl radical scavenging activity of coffee beans. Briefly, 50 μl sample extract, 50 μl 6 mM ferrous sulfate heptahydrate, and 50 μl 6 mM hydrogen peroxide were injected into plate and incubated for 10 min under about 25°C. Subsequently, 50 μl 6 mM 3‐hydroxybenzoic acid was added. Trolox (0–400 μg/ml) and water were used for calibration and blank, respectively. The absorbance was measured at 510 nm and the results were expressed as mg TE/g ± SD.

#### 2.4.8 Estimation of ferrous ion chelating activity (FICA)

Modified FICA assay was performed according to the method of Dinis et al. (1994) with some modifications. Briefly, 15 μl sample extract or EDTA standard, 85 μl water, 50 μl 2 mM ferrous chloride, and 50 μl 5 mM ferrozine were injected into plate and incubated for 10 min in the dark at 25°C. EDTA (0–50 μg/ml) and water were used for calibration curve and blank, respectively. The absorbance was measured at 562 nm and the results were expressed as mg EDTA equivalents per dry weight (mg EE/g) ± SD.

#### 2.4.9 Estimation of reducing power (RPA)

Modified RPA assay according to the method of Ferreira et al. (2007) was used for the evaluation of the reducing power of coffee beans with the color changing from yellow to green. Briefly, 10 μl sample extract, 25 μl 0.2 M phosphate buffer (pH 6.6), and 25 μl 1% potassium ferricyanide (III) solution were injected into plate followed by 20 min incubation at 25°C. Subsequently, 25 μl 10% trichloroacetic acid was added to stop the reaction followed by the addition of 85 μl water and 8.5 μl 0.1% ferric chloride solution, and 15 min incubation at 25°C. Trolox (0–500 μg/ml) and water were used for calibration curve and blank, respectively. The absorbance was measured at 750 nm and the results were expressed as mg TE/g ± SD.

### 2.5 Characterization of phenolic compounds via LC‐ESI‐QTOF‐MS/MS

The characterization of phenolic compounds is analyzed via the LC‐ESI‐QTOF‐MS/MS method of Peng et al. (2019) with some modifications. Agilent 1200 series HPLC combined with Agilent 6520 Accurate‐Mass Q‐TOF LC/MS through an electrospray ionization source (ESI) was used for preliminary identification and characterization. Separation of extracts (10°C) was carried out on a Synergi Hydro‐RP 80A, LC column (250 mm × 4.6 nm, 4 µm) (Phenomenex, Lane Cove, NSW, Australia), at 25°C. Mobile phase A is 98% acetic acid in water and mobile phase B is the mixture of acetonitrile, water, and acetic acid at the ratio of 100:1:99 (v/v/v). Obtaining the mass spectra in the *m/z* range 50 to 1300 and identifying peaks in both positive and negative ionization modes. The acquisition and analysis of all data are conducted by Mass Hunter Data Acquisition Software Version B.03.01 and Personal Compounds Database and Library (PCDL). Further MS/MS identification and *m/z* characterization are required if the mass error of selected compounds is lower than 5 ppm and the score of PCDL is over 80.

### 2.6 Identification and quantification of volatile compounds by headspace/gas chromatography–mass spectrometry (HS‐SPME‐GC‐MS)

Volatile compounds in coffee ground samples were analyzed by HS‐SPME‐GC‐MS according to the method of Rocchetti et al. (2020). GC‐MS analysis was conducted via a gas chromatograph (6850 series II Network GC System, Agilent Technologies, USA) coupled to an HS‐SPME system (PAL RSI I20, Switzerland) and a mass spectrometer (5973Network Mass Selective Detector, Agilent Technologies, USA). A 30‐m DB‐Wax capillary column (Agilent Technologies, USA) with 0.25 mm internal diameter and 0.25 µm film thickness was chosen with the combination of 65 μm PDMS/DVB fiber (Fused Silica, Sigma Aldrich, USA). The carrier gas was helium with 60 kPa column head pressure. Samples were incubated for 15 min at 60°C, then 15 min extraction and 6 min desorption. The GC oven program was set as follow: 40°C for 5 min followed by an increase to 190°C with the rate of 5°C/min for 8 min; subsequently, the temperature reached 240°C at a rate of 10°C/min and maintained for 10 min. The acquisition was in SCAN mode (35–350 m*/z*). The solvent delay time was 2 min.

One gram ground coffee sample mixed with 20 μl 100 mg/L 4‐Octanol as internal standard was added into vials and then injected as the temperature gradient program above. The linear retention index (LRI) was calculated by alkane standard (C_7_–C_20_) as the following equation that compares the retention time of one target compound (RT_x_) with those of n‐alkanes with *n* and *n* + 1 carbon eluted before and after the target compound (RT_n_):

$$\mathrm{LRI}\;\left(\mathrm{target}\;\mathrm{compound}\right)=100\times \frac{{\mathrm{RT}}_{x}‐{\mathrm{RT}}_{n}}{{\mathrm{RT}}_{n+1}‐{\mathrm{RT}}_{n}}+n$$

LRI and mass spectrum of volatile compounds detected in coffee samples were compared to the data in the NIST Chemistry WebBook spectrum library (NIST2017) and NIST mass spectra database, respectively. Semi‐quantification was conducted by comparing the response area of the target compound and a closely eluted compound with known concentration after LRI and compound MS confirmed.

### 2.7 Statistical analysis

All results were pure results subtracted by blanking or control values and expressed as mean ± standard deviations (SD) of triple independent analyses. All the statistical analysis was conducted by Minitab 19 (Minitab^®^ for Windows Release 19, Minitab Inc., Chicago) and GraphPad Prism 9. One‐way analysis of variance (ANOVA) and Tukey's honestly significant differences (HSD) were used to verify and analysis the significant differences among samples.

## 3 RESULTS AND DISCUSSION

### 3.1 Phenolic content estimation (TPC, TFC, and tannins content)

The results of TPC, TFC, and TCT for the estimation of phenolic content in the coffee beans were analyzed as shown in Table 1. Overall, all values of the free phenolic compounds were higher than that of the bound, except TCT. There were significant differences (*p* < .05) shown in the phenolic content of coffee beans with different roasted degrees.

**TABLE 1:** Determination of phenolic content in coffee beans with three roasting degrees and their antioxidant activity

Antioxidant assays	Light roasting	Medium roasting	Dark roasting
Free Phenolic			
TPC (mg GAE/g)	23.97 ± 0.60^a^	22.41 ± 0.58^b^	20.14 ± 0.72^c^
TFC (mg QE/g)	0.97 ± 0.01^a^	0.87 ± 0.02^b^	1.16 ± 0.04^b^
TCT (mg CE/g)	1.87 ± 0.23^a^	3.51 ± 0.02^b^	5.46 ± 0.21^c^
DPPH (mg TE/g)	148.55 ± 6.28^a^	147.86 ± 5.50^a^	143.32 ± 2.59^a^
FRAP (mg TE/g)	30.34 ± 1.03^a^	27.63 ± 0.89^b^	29.50 ± 0.52^a^
ABTS (mg TE/g)	101.72 ± 1.05^a^	102.37 ± 8.10^a^	94.87 ± 5.10^a^
^·^OH‐RSA (mg TE/g)	18.72 ± 0.10^c^	22.41 ± 2.17^b^	32.94 ± 0.29^a^
FICA (mg EE/g)	0.37 ± 0.09^a^	0.49 ± 0.02^a^	0.51 ± 0.03^a^
RPA (mg TE/g)	48.06 ± 4.75^a^	42.33 ± 1.44^b^	52.18 ± 1.38^a^
Bound Phenolic			
TPC (mg GAE/g)	19.32 ± 1.29^a^	17.86 ± 0.04^b^	15.83 ± 1.28^c^
TFC (mg QE/g)	0.935 ± 0.04^a^	0.71 ± 0.04^b^	0.74 ± 0.07^b^
TCT (mg CE/g)	12.01 ± 0.17^a^	6.39 ± 0.48^b^	2.10 ± 0.01^c^
DPPH (mg TE/g)	69.98 ± 2.26^b^	75.80 ± 0.48^a^	77.39 ± 0.89^a^
FRAP (mg TE/g)	17.95 ± 0.84^a^	18.13 ± 0.15^a^	17.40 ± 0.13^a^
ABTS (mg TE/g)	61.51 ± 2.20^a^	69.51 ± 4.20^a^	64.62 ± 4.84^a^
^·^OH‐RSA (mg TE/g)	36.85 ± 0.26^b^	50.63 ± 7.31^a^	52.11 ± 2.07^a^
FICA (mg EE/g)	3.61 ± 0.25^a^	3.39 ± 0.17^a^	3.28 ± 0.08^a^
RPA (mg TE/g)	9.20 ± 1.89^b^	17.01 ± 1.33^a^	15.80 ± 3.34^a^

Note

Values expressed as mean ± standard deviation per gram dry weight. Values within the same rows with different superscript letters (^a,b,c^) indicate that they are significantly different from each other (*p* < .05).

Abbreviations: ^·^OH‐RSA, hydroxyl radical scavenging activity; ABTS, 2,2’‐azino‐bis‐3‐ethylbenzothiazoline‐6‐sulfonic acid assay; CE, catechin equivalents; DPPH, 2,2’‐diphenyl‐1‐picrylhydrazyl assay; EDTA, ethylenediaminetetraacetic acid; FICA, ferrous ion chelating activity; FRAP, ferric reducing antioxidant power assay; GAE, gallic acid equivalents; QE, quercetin equivalents; RPA, reducing powder assay; TAC, total antioxidant capacity; TE, Trolox equivalents; TFC, total flavonoid content; TPC, total phenolic content; TTC, total tannin content.

In terms of TPC, the light‐roasted coffee beans possessed the highest value with 23.97 ± 0.60 mg GAE/g, followed by medium (22.41 ± 0.58 mg GAE/g) and dark roasted (20.14 ± 0.72 mg GAE/g). It is consistent with previous research that the decreased tendency of the total content of phenolic compounds is along with the intensification of roasting (Cho et al., 2014; Król et al., 2020; Somporn et al., 2011). Polyphenolic compounds, especially chlorogenic acids in coffee beans, performing highly thermal instability, could be directly decomposed with a temperature higher than 80°C which cause the TPC reduction after intensive roasting (Hecimovic et al., 2011; Król et al., 2020). Partial bound phenolic compounds existing in the plant matrix could be liberated during thermal processing by disrupting cellulose constituents (Cho et al., 2014; Mehari et al., 2020; Somporn et al., 2011). With the comparison to bound TPC values, it contributes to the accumulation of free phenolic compounds, which avoids the huge decrease but is relatively higher in the free TPC values. Furthermore, a similar trend and significant differences were shown in the bound TPC value that the total bound phenolic compounds decreased from 19.32 ± 1.29 mg GAE/g to 15.83 ± 1.28 mg GAE/g with the increasing roasting degree. However, the existence of Maillard reaction products, especially melanoidins, has to be considered because of its interaction with Folin–Ciocalteu reagent, which could increase the value of total phenols (TPC) to some extent (Pérez‐Hernández et al., 2012). It is necessary to cross‐analyze through other antioxidant assays to estimate the properties.

When it comes to TFC and TCT, comparing to the bound phenolic, the results of free phenolic compounds exhibited a reverse trend that the free TFC and TCT values increased from 0.97 ± 0.01 mg QE/g and 1.87 ± 0.23 mg CE/g to 1.16 ± 0.04 mg QE/g and 5.46 ± 0.21 mg CE/g with significant differences when roasting degree increased. Similar results were observed by Hecimovic et al. (2011), Odzakovic et al. (2016) and Król et al. (2020) that the content of total flavonoids and tannins was directly proportional to the roasting degree. More and more bound phenolic compounds were released by the increasing roasting temperature, which improved the free TFC and TCT while reduced that of the bound reasonably. Suitable roasting could degrade condensed tannins into the lower molecular mass of flavonoids, such as anthocyanin, which could improve free TFC value to some extent. However, tannins perform high thermal resistance whose content could be slightly reduced with the temperature lower than 210°C (Ahmad et al., 2018; Van Cuong et al., 2014). Flavan‐3‐ol is the monomer of condensed tannins which belongs to flavonoids, whereas gallic acid is a component of hydrolyzable tannins belonging to nonflavonoids (Mehari et al., 2020). Thus, various compounds including flavan‐3‐ols complexes, quinolactones, and gallic acids complexes could be formed via the isomerization and polymerization of polyphenolic compounds and interactions with proteins and sugars during thermal processing, which could induce the increase in TPC and TCT (Farah & Donangelo, 2006; Hecimovic et al., 2011; Kim et al., 2011; Król et al., 2020).

### 3.2 LC‐ESI‐QTOF‐MS/MS characterization of phenolic compounds in roasted coffee beans

The phenolic compounds in roasted coffee beans including phenolic acids and flavonoids were untargeted and tentatively identified and characterized according to their *m/z* value and MS spectra through both negative and positive modes of ionization. The compounds with not only mass error less than 6 ppm but also PCDL library scores higher than 80 were selected for further MS/MS analysis and verification.

#### 3.2.1 Distribution of detected phenolic compounds—Venn diagram

**FIGURE 1:**
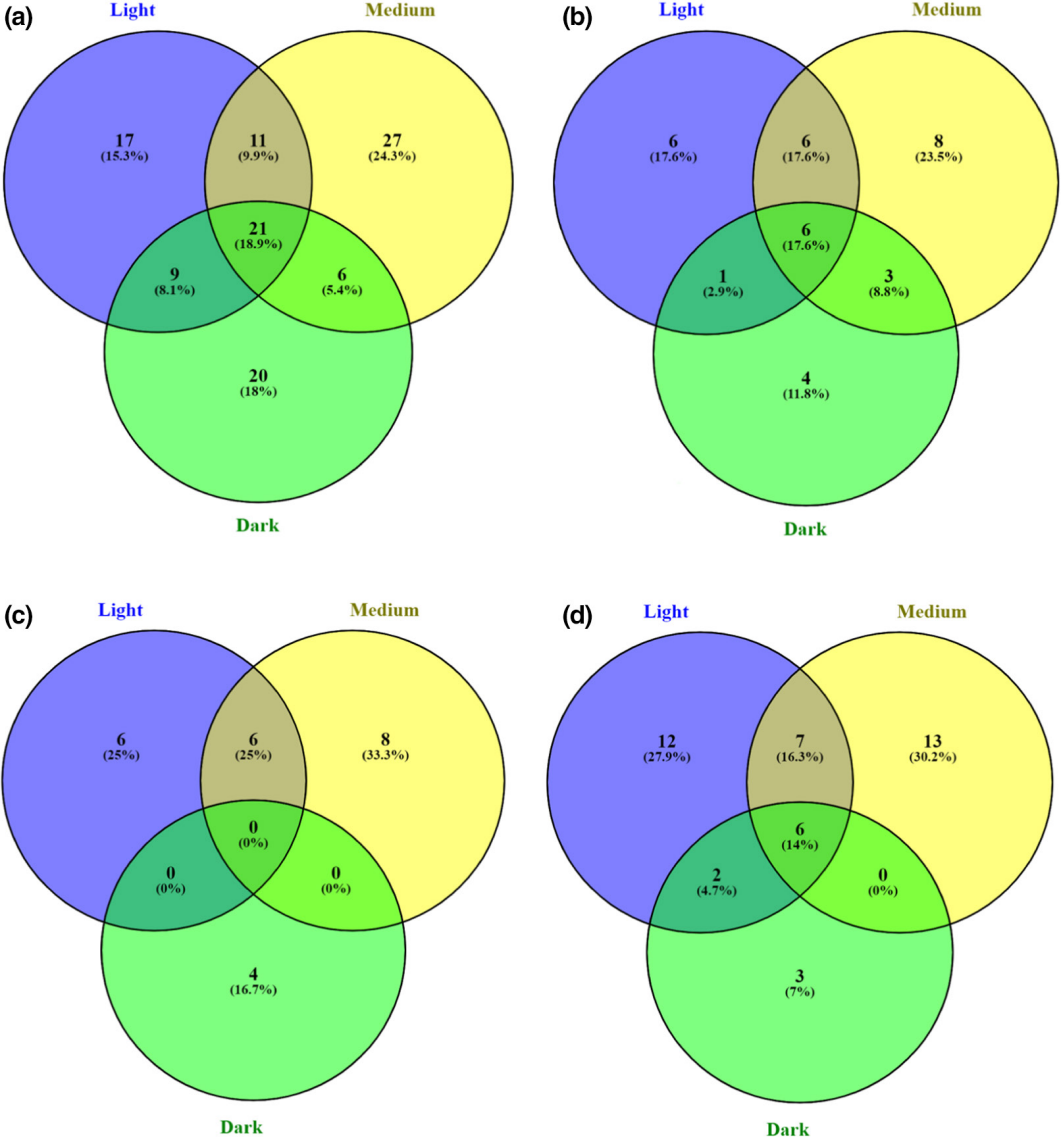
Venn diagram of phenolic compounds present in roasted coffee samples. The similarity of total phenolic compounds (a), phenolic acids (b), flavonoids (c), and other phenolic compounds (d) profiles between the coffee beans with three roasting levels

According to Figure 1a, a total of 111 phenolic compounds were identified in coffee beans with three roasted levels. There were 18.9% phenolic compounds presented in all roasted samples. Medium‐roasted coffee beans contained the highest proportion of unique phenolic compounds (24.3%), followed by the dark‐ (18%) and the light‐roasted samples (15.3%). Light‐roasted coffee beans shared the highest proportion of common characterized compounds with the medium roasted (9.9%), followed by the dark roasted (8.1%), whereas the percentage of common identified phenolic compounds between the medium‐ and the dark‐roasted coffee beans is the lowest at 5.4%. As shown in Figure 1b, 17.6% of phenolic acids were common in all roasted coffee beans. Medium‐roasted beans had the highest proportion of unique phenolic acids with 23.5%, followed by the light and dark. Interestingly, there were no common flavonoids shared in all samples with the observation of Figure 1c. The dark‐roasted coffee beans performed a completely different flavonoids composition from the light‐ and medium‐roasted coffee beans. A similar situation could be observed from Figure 1d that the light‐ and medium‐roasted coffee beans had similar other phenolic compounds profile, whereas the dark samples exhibited significant differences.

The Venn graph indicates that reasonable roasting could improve the generation of various phenolic compounds owing to the degradation of free phenolic compounds and the releasement of bond phenolic compounds properly (Cho et al., 2014; Król et al., 2020; Mehari et al., 2020; Somporn et al., 2011). However, the existed and generated phenolic compounds would be degraded and decomposed with the continual intensification of thermal processing so that the proportion of phenolic compounds decreased in the dark‐roasted coffee beans (Hecimovic et al., 2011; Król et al., 2020). The influence on the specific phenolic compounds and their changes would be further investigated.

#### 3.2.2 LC‐MS/MS‐based characterization of phenolic compounds

Totally, 23 phenolics in three different roasted coffee beans were identified and characterized, which were mainly adopted by 15 phenolic acid and 5 other polyphenols.

##### Phenolic acids

Five different subclasses of phenolic acids were characterized in the light‐, medium‐, and dark‐roasted coffee beans as shown in Table 2, which primarily consists of hydroxycinnamic acid derivatives. The derivatives of hydroxybenzoic acids, hydroxyphenylpropanoic acids, hydroxyphenylpentanoic acids, and hydroxyphenylacetic acids have also been traced in different roasted coffee beans, especially the medium roasted.

**TABLE 2:** Characterization of phenolic compounds in different roasted coffee beans by LC‐ESI‐QTOF‐MS/MS

No.	Proposed compounds	Molecular formula	RT (min)	Ionization (ESI^+^/ESI^−^)	Molecular weight	Theoretical (*m/z*)	Observed (*m/z*)	Error (ppm)	MS^2^ product ions	Coffee beans
Phenolic acid										
Hydroxybenzoic acids										
1	2,3‐Dihydroxybenzoic acid	C_7_H_6_O_4_	14.815	[M−H]^−^	154.0266	153.0193	153.0198	3.3	109	M
2	2‐Hydroxybenzoic acid	C_7_H_6_O_3_	18.259	**[M−H]^−^	138.0317	137.0244	137.0249	3.6	93	*M, L, D
Hydroxycinnamic acids										
3	Caffeoyl tartaric acid	C_13_H_12_O_9_	4.064	[M+H]^+^	312.0481	313.0554	313.0563	2.9	161	*M, L, D
4	Ferulic acid	C_10_H_10_O_4_	15.728	[M−H]^−^	194.0579	193.0506	193.0502	−2.1	178, 149, 134	*M, L
5	Ferulic acid 4‐*O*‐glucoside	C_16_H_20_O_9_	17.403	**[M−H]^−^	356.1107	355.1034	355.1042	2.3	193, 178, 149, 134	*M, D
6	3‐Feruloylquinic acid	C_17_H_20_O_9_	18.729	[M−H]^−^	368.1107	367.1034	367.1043	2.5	298, 288, 192, 191	*L, M
7	3‐Caffeoylquinic acid	C_16_H_18_O_9_	20.622	**[M−H]^−^	354.0951	353.0878	353.0887	2.5	253, 190, 144	*M, L, D
8	Caffeic acid	C_9_H_8_O_4_	21.831	[M−H]^−^	180.0423	179.035	179.0356	3.4	143, 133	M
9	3‐*p*‐Coumaroylquinic acid	C_16_H_18_O_8_	22.008	**[M−H]^−^	338.1002	337.0929	337.0938	2.7	265, 173, 162	*M, L
10	1,5‐Dicaffeoylquinic acid	C_25_H_24_O_12_	31.498	**[M−H]^−^	516.1268	515.1195	515.1211	3.1	353, 335, 191, 179	*L, M, D
11	*p*‐Coumaroyl glycolic acid	C_11_H_10_O_5_	37.342	[M+H]^+^	222.0528	223.0601	223.0604	1.3	163	*D, M
Hydroxyphenylpropanoic acids										
12	3‐Hydroxy‐3‐(3‐hydroxyphenyl) propionic acid	C_9_H_10_O_4_	10.956	**[M−H]^−^	182.0579	181.0506	181.05	−3.3	163, 135, 119	D
13	Dihydroferulic acid 4‐*O‐*glucuronide	C_16_H_20_O_10_	11.159	[M−H]^−^	372.1056	371.0983	371.0983	0.0	195	M
Hydroxyphenylpentanoic acids										
14	5‐(3'‐Methoxy‐4'‐hydroxyphenyl)‐*ᵞ* ‐valerolactone	C_12_H_14_O_4_	31.143	[M+H]^+^	222.0892	223.0965	223.0954	−4.9	205	M
Hydroxyphenylacetic acids										
15	2‐Hydroxy‐2‐phenylacetic acid	C_8_H_8_O_3_	20.229	**[M−H]^−^	152.0473	151.04	151.0405	3.3	136, 92	*L, M, D
Flavonoids										
Flavonols										
16	3‐Methoxysinensetin	C_21_H_22_O_8_	16.528	**[M+H]^+^	402.1315	403.1388	403.1395	1.7	388, 373, 355, 327	L
Other polyphenols										
Hydroxybenzaldehydes										
17	p‐Anisaldehyde	C_8_H_8_O_2_	13.709	**[M+H]^+^	136.0524	137.0597	137.0599	1.5	122, 109	*M, L
18	4‐Hydroxybenzaldehyde	C_7_H_6_O_2_	22.552	**[M−H]^−^	122.0368	121.0295	121.0297	1.7	77	L
Hydroxycinnamaldehydes										
19	Ferulaldehyde	C_10_H_10_O_3_	24.716	[M−H]^−^	178.063	177.0557	177.0567	5.6	161, 147	M
Curcuminoids										
20	Curcumin	C_21_H_20_O_6_	4.095	[M−H]^−^	368.126	367.1187	367.1177	−2.7	217	L
Tyrosols										
21	Demethyloleuropein	C_24_H_30_O_13_	44.294	[M−H]^−^	526.1686	525.1613	525.1595	−3.4	495	M
Lignans										
22	Sesamin	C_20_H_18_O_6_	3.984	[M−H]^−^	354.1103	353.103	353.102	−2.8	338, 163	L
Stilbenes										
23	Resveratrol	C_14_H_12_O_3_	31.317	**[M−H]^−^	228.0786	227.0713	227.0709	−1.8	212, 185, 157, 143	D

Note

Ionization mode with ** represents that the compound was detected in both positive and negative modes but only one mode's data were presented. For compounds found in more than one sample, only results for samples with * were shown in the table. Roasted coffee beans samples mentioned in abbreviations are Light roasted “L”, Medium roasted “M”, and Dark roasted “D.”

Abbreviation: RT, retention time.

###### Hydroxybenzoic acids

Compound 1 with [M−H]^−^ ion *m/z* at 153.0193 detected only in the medium‐roasted coffee beans extract was tentatively characterized as 2,3‐dihydroxybenzoic. However, another compound (retention time (RT) = 18.259 min), 2‐hydroxybenzoic acid, detected in both negative and positive mode with [M−H]^−^ ion at *m/z* 137.0244, was presented in all roasted coffee samples. Two compounds classified in hydroxybenzoic acids performed product ions at *m/z* 109 and at *m/z* 93, which represented the loss of CO_2_ (44 Da) from the precursor ions (Saez et al., 2019; Suleria et al., 2020).

Our detection agreed with previous research conducted by Moreira et al. (2017), Gorecki and Hallmann (2020), and Król et al. (2020). Moreira et al. (2017) discovered 2,3‐dihydroxybenzoic and 2‐hydroxybenzoic acid in both roasted Arabica and Robusta coffee beans. 2‐hydroxybenzoic acid is known as salicylic acid which has also been discovered in roasted coffee beans by Gorecki and Hallmann (2020) and Król et al. (2020). During roasting, chlorogenic acids, as the main component of phenolic fraction in coffee beans, would be hydrolyzed into various aromatic metabolites including salicylic acid (Gorecki & Hallmann, 2020; Król et al., 2020). Therefore, the content of salicylic acid in coffee beans would be increased along with the intensification of roasting within certain temperatures. Previously, several other derivatives of hydroxybenzoic acids including *p*‐hydroxybenzoic, syringic, and 2,4‐dihydroxybenzoic acid were detected in canned coffee drink, honey, and iced tea as well (Shalash et al., 2017; Zeb, 2021).

###### Hydroxycinnamic acids

In our study, hydroxycinnamic acids contained collectively a larger number of detected compounds than in any other subclass. Ferulic acid and caffeic acid were observed with [M−H]^−^ *m/z* at 15.728 and 21.831 in the negative ionization mode only. The identification of ferulic acid was confirmed through the fragments at *m/z* 178, *m/z* 149, and *m/z* 134, which indicated the loss of CH_3_, CO_2_, and CH_3_ with CO_2_ from the precursor, respectively. The fragmentation in MS^2^ spectrum of caffeic acid performed the product ions of *m/z* 143 and *m/z* 133, which referred to the loss of two water and HCOOH. Ferulic acid is a natural antioxidant that can be found in not only coffee beans but also various fruits (orange and grapefruit), vegetables (broccoli and tomato), and coffee beans (Kaur et al., 2018; Król et al., 2020). Previously, ferulic acid has also been detected in roasted coffee beans by Moreira et al. (2017). Ameca et al. (2018) reported that ferulic acid was relatively higher in concentration in the fermented coffee cherry pulp but suddenly decreased after drying. However, Shalash et al. (2017) reported a significantly low concentration of ferulic acid detected in canned coffee drinks and even no trace in iced tea. Król et al. (2020) did not detect it in both coffee beans after fresh roasting nor after 1 year of storage neither. Different from ferulic acid, caffeic acid has been commonly detected in roasted coffee beans. According to Somporn et al. (2011) and Król et al. (2020), the level of caffeic acid rapidly increased when the roasting temperature increased within 230 to 250°C owing to the decomposition of chlorogenic acids. It agrees with our results that both compounds were detected in the medium‐roasted coffee beans but no trace in the light and dark roasted.

3‐feruloylquinic acid (compound 6), 3‐caffeoylquinic acid (compound 7), and 3‐*p*‐coumaroylquinic acid (compound 9) with [M−H]^−^ ion *m/z* at 367.1034, 353.0878, and 337.0929 were all detected in the medium‐roasted coffee beans. The presence of 3‐FQA was identified by the product ions of *m/z* 298 [M−H^−^ 3H_2_O_2_‐CH_3_, loss of 69 Da], *m/z* 288 [M−H^−^ H_2_O‐CH_3_‐HCOOH, loss of 79 Da], *m/z* 192 [M−H^−^ C_7_H_11_O_5_, loss of 175 Da], and *m/z* 191 [M−H^−^ C_10_H_8_O_3_, loss of 176 Da] (Lin et al., 2019; Suleria et al., 2020). The identity of 3‐CQA has confirmed fragments at *m/z* 253, *m/z* 190, and *m/z* 144, which represented the loss of HCOOH‐3H_2_O (100 Da), C_6_H_5_O_2_‐3H_2_O (163 Da), and C_7_H_11_O_6_‐H_2_O (209 Da) from the parent ion (Lin et al., 2019; Suleria et al., 2020).

3‐feruloylquinic acid and 3‐caffeoylquinic acid, also known as 3‐FQA and 3‐CQA, are the two typical isomers of caffeoylquinic acid and feruloylquinic acid, which are the two primary subclass of chlorogenic acids in the coffee beans (Monteiro et al., 2007; Rostagno et al., 2015). As the esterified product of quinic acid and trans‐cinnamic acid derivatives, chlorogenic acids continually degrade along with the roasting period which allows the generation of these phenolic compounds, especially 3‐CQA (Rostagno et al., 2015). Khamitova et al. (2020) also claimed that 3‐CQA and 5‐CQA were the major cinnamoyl ester in both Arabica and Robusta coffee beans, meanwhile Heeger et al. (2017) emphasized that 3‐CQA took the significantly high proportion in the coffee cherry pulp. Both compounds have been detected in roasted coffee beans previously by Monteiro et al. (2007) and Moreira et al. (2017) as well. Similarly, 3‐*p*‐coumaroylquinic acid is also recognized as 3‐*p*CoQA which is the isomer belonging to the main subclass of chlorogenic acids and *p*‐Coumaroylquinic acids (*p*CoQAs). Ortiz (2017) and Badmos et al. (2019) detected 3‐*p*CoQA and other isomers in both Arabica and Robusta coffee beans, whereas Ortiz (2017) claimed that the level of *p*CoQAs performed significantly lower than CQA and FQA in roasted coffee beans.

###### Hydroxyphenylpropanoic, hydroxyphenylpentanoic, and hydroxyphenylacetic acids

For hydroxyphenylpropanoic acids, 3‐Hydroxy‐3‐(3‐hydroxyphenyl)propionic acid (compound 12) with [M−H]^−^ ion *m/z* at 181.0506 and dihydroferulic acid 4‐*O*‐glucuronide (compound 13) with [M−H]^−^ ion *m/z* at 181.0506 were characterized and detected in only the dark‐roasted and medium‐roasted coffee beans, respectively. Madrid‐Gambin et al. (2016) detected the 3‐Hydroxy‐3‐(3‐hydroxyphenyl)propionic acid (HPHPA) in a coffee beverage with a rich chlorogenic acids extract. They also discovered that the level of HPHPA would be increased in urine after sustained intake of coffee (Madrid‐Gambin et al., 2016). Previously, Guertin et al. (2015) also detected and observed a similar situation. Thus, Madrid‐Gambin et al. (2016) discussed that HPHPA could be the metabolites of chlorogenic acids by microbiota and formed by the reduction in the double bond of caffeic acid (Ludwig et al., 2013). The identification of dihydroferulic acid 4‐*O*‐glucuronide was confirmed by the product ions at *m/z* 195, which indicated the loss of glucuronide (176 Da) moiety from precursor ions (Sasot et al., 2017; Suleria et al., 2020). Similarly, the trace of dihydroferulic acid 4‐*O*‐glucuronide was discovered in human urine after the consumption of coffee by Stalmach et al. (2009) but with nonquantifiable amounts in plasma.

When it comes to two detected compounds belonging to hydroxyphenylpentanoic and hydroxyphenylacetic acids, there is no detection in coffee beans and drinks in the previous studies. 2‐Hydroxy‐2‐phenylacetic acid is also known as mandelic acid which is usually present in fruit seeds, such as apple and papaya, and nuts including walnuts, almond, and sunflower seeds (Przybylska‐Balcerek & Stuper‐Szablewska, 2019).

##### Flavonoids—Flavonols

The only flavonoid detected in only light‐roasted coffee beans was 3‐methoxysinensetin, a polymethoxyflavone, with [M−H]^−^ ion *m/z* at 403.138. The identification of 3‐methoxysinensetin was confirmed based on the MS^2^ fragmentation with the product ions at *m/z* 388, *m/z* 373, *m/z* 355, and *m/z* 134 (Suleria et al., 2020). Previously, there is no detection of 3‐methoxysinensetin in roasted coffee beans. However, Chiari‐Andréo et al. (2017) observed 3‐methoxysinensetin in fresh guava and Li et al. (2006) also detected it in the sweet orange (*Citrus sinensis*) peel.

##### Other polyphenols

As for other polyphenols, four subclasses in roasted coffee beans have been characterized, which include two hydroxybenzaldehydes, one hydroxycinnamaldehydes, one curcuminoid, and one tyrosol. All five compounds were not detected in the dark‐roasted coffee beans in our study.

Compound 18 was tentatively identified as 4‐hydroxybenzaldehyde based on the precursor ion at both positive and negative mode with *m/z* at 121.0295 and confirmed through the product ion at *m/z* 77, which indicated the loss of CO_2_ from the precursor (Suleria et al., 2020). 4‐hydroxybenzaldehyde is one of the major fragrance and flavor components of natural vanilla which is usually used as a flavoring for coffee and chocolate owing to its aromatic properties (Linares et al., 2019). Mahmud et al. (2020) discovered the presence of 4‐hydroxybenzaldehyde in roasted coffee, meanwhile Lazzari et al. (2019) did not detect its trace in the spent coffee grounds but observed in rice husk, peanut shell, and peach core. Narváez‐Cuenca et al. (2020) also found it in the edible extract oil of guava seeds.

Ferulaldehyde (compound 19) was detected only in the medium‐roasted coffee beans which is also known as 4‐hydroxy‐3‐methoxycinnamaldehyde. Previously, it was also discovered in the coffee drink by Xu et al. (2019). Moreover, Rojas‐Padilla and Vásquez‐Villalobos (2016) traced ferulaldehyde in potato but with lower concentration. Curcumin (compound 20) was discovered only in the light‐roasted coffee beans in the negative mode with [M−H]^−^ ion *m/z* at 367.1187. Its identification was confirmed by the fragmentation with the product ion at *m/z* 217, which indicated the loss of C_9_H_10_O_2_ (150 Da) from the precursor ions (Suleria et al., 2020). Curcumin is the major substance of turmeric and also can be detected in the herbal remedy (Sharma et al., 2005; Soleimani et al., 2018). Traditionally, curcumin is used as a spice and coloring agent in most cuisines or for therapeutic applications, such as anti‐inflammatory and antimicrobial activities (Mohajeri et al., 2018; Soleimani et al., 2018). Demethyloleuropein (compound 21) plays a significant role as a protector and natural bioactive component and is commonly discovered in olive fruits (Sivakumar et al., 2007). Previously, the track of both curcumin and demethyloleuropein in roasted coffee beans has not been reported.

##### Lignans and stilbenes

Sesamin (Compound 22) is the only lignans detected in the light‐roasted coffee beans in our study with [M−H]^−^ ion *m/z* at 353.103. Sesamin is the primary lignin that could be discovered from sesame seeds and sesame oil. It is also present in flax, barley, buckwheat, millet, oats, rye, nuts, and legumes (Dalibalta et al., 2020). Previously, Sesamin has been isolated from sesame by Majdalawieh et al. (2020). However, the presence of sesamin has not been reported in roasted coffee beans.

Differently, resveratrol (compound 23) is only detected in the dark‐roasted coffee beans with [M−H]^−^ ion *m/z* at 227.0713. Our result was similar with Saeed Alkaltham et al. (2020), who detected the presence of resveratrol in both green and roasted coffee beans and claimed that roasting could improve the level of resveratrol within a certain range. Ramon‐Goncalves et al. (2019) observed its trace in Arabica, Portuguese coffee beans, and their coffee residue. Resveratrol could also be discovered and isolated from grapes as well (Roat & Saraf, 2017; Sasot et al., 2017).

### 3.3 Volatile compounds in different roasted coffee beans

The composition of volatile compounds in two types of coffee beans with different roasting degrees analyzed by the HS‐SPME‐GC‐MS method was identified as shown in Table 3. The content of primary volatile compounds in coffee beans all was improved along with the intensive roasting degree, particularly acetic acids, furans, and furanic compounds, and some heterocyclic nitrogen compounds, which is consisted with the previous research (Caporaso et al., 2018; Hertz‐Schunemann et al., 2013; Somporn et al., 2011). Acetic acid was the most abundant organic acid in roasted coffee beans after roasting, which is probably because of the fragmentation of saccharides, especially sucrose (Diviš et al., 2019). During roasting, the hydrolysis of sucrose with the evaporation of residual water could produce fructose which could generate 2,3‐endiol via Lobry‐de‐Bruynvan‐Eckenstein rearrangement. Thermal dehydration of these sugars would form 1‐deoxyglucosone as an acid precursor which could induce the formation of acetic acid eventually (Ginz et al., 2000; Yeretzian et al., 2014).

**TABLE 3:** The content of volatile compounds identified in different roasted coffee beans by HS‐SPME‐GC‐MS

Comp no.	Compound name	Molecular formula	RT (min)	LRI	LRI (NIST)	Content (μg/g)		
						Light roasted	Medium roasted	Dark roasted
Pyridines								
1	Pyridine	C_5_H_5_N	10.63	1175	1169	1.88 ± 0.03	2.93 ± 0.03	5.31 ± 0.10
Pyrazines								
2	Pyrazine	C_4_H_4_N_2_	11.48	1201	1201	0.56 ± 0.02	0.625 ± 0.03	0.85 ± 0.06
3	Pyrazine, methyl‐	C_5_H_6_N_2_	13.18	1255	1252	6.25 ± 0.01	6.17 ± 0.01	7.03 ± 0.04
4	Pyrazine, 2,5‐dimethyl‐	C_6_H_8_N_2_	14.90	1311	1318	3.75 ± 0.04	3.48 ± 0.15	3.23 ± 0.02
5	Pyrazine, 2,6‐dimethyl‐	C_6_H_8_N_2_	15.09	1318	1314	2.89 ± 0.04	2.45 ± 0.05	2.50 ± 0.02
6	Pyrazine, ethyl‐	C_6_H_8_N_2_	15.22	1322	1325	0.57 ± 0.02	0.59 ± 0.01	0.63 ± 0.01
7	Pyrazine, 2‐ethyl‐6‐methyl‐	C_7_H_10_N_2_	16.73	1374	1375	0.52 ± 0.01	0.52 ± 0.01	0.52 ± 0.01
Acids and esters								
8	Acetic acid	C_2_H_4_O_2_	18.40	1435	1434	52.66 ± 0.70	60.4 ± 0.46	60.83 ± 3.36
9	2‐Butenoic acid, 3‐methyl‐	C_5_H_8_O_2_	26.83	1791	1802	1.53 ± 0.20	1.52 ± 0.22	1.48 ± 0.21
Furan and Furanic compounds								
10	Furfural	C_5_H_4_O_2_	18.72	1447	1443	16.26 ± 0.36	17.44 ± 0.16	19.32 ± 0.37
11	2‐Furanmethanol, acetate (furfuryl acetate)	C_7_H_8_O_3_	20.73	1525	1523	0.99 ± 0.31	1.06 ± 0.09	1.60 ± 0.49
12	2‐Furancarboxaldehyde, 5‐methyl‐ (5‐Methylfurfural)	C_6_H_6_O_2_	21.53	1558	1555	14.16 ± 0.27	17.60 ± 0.43	18.93 ± 0.42
13	2‐Furanmethanol (Furfuryl alcohol)	C_5_H_6_O_2_	23.78	1654	1658	34.16 ± 0.40	37.87 ± 0.31	42.97 ± 1.65
Pyrrole								
14	1H‐Pyrrole‐2‐carboxaldehyde, 1‐methyl‐	C_6_H_7_NO	22.68	1605	1607	0.65 ± 0.01	0.83 ± 0.04	1.03 ± 0.04
Ketones								
15	2‐Cyclopenten‐1‐one, 2‐hydroxy‐3‐methyl‐	C_6_H_8_O_2_	27.55	1824	1827	1.15 ± 0.16	1.48 ± 0.12	1.90 ± 0.18
16	2‐Cyclopenten‐1‐one, 3‐ethyl‐2‐hydroxy‐	C_7_H_10_O_2_	28.98	1888	1894	0.72 ± 0.11	1.17 ± 0.29	0.935 ± 0.13
17	Ethanone, 1‐(1H‐pyrrol‐2‐yl)‐	C_6_H_7_NO	30.51	1957	1957	1.60 ± 0.57	2.01 ± 0.10	2.48 ± 0.41
Phenols								
18	Maltol	C_6_H_6_O_3_	30.43	1953	1954	0.83 ± 0.27	1.31 ± 0.38	1.97 ± 0.77
19	Phenol	C_6_H_6_O	31.17	1986	1989	1.01 ± 0.03	1.11 ± 0.08	1.52 ± 0.04
Other compounds								
20	Anethole	C_10_H_12_O	27.44	1819	1818	1.22 ± 0.70	1.50 ± 0.71	1.62 ± 1.29

Abbreviations: LRI, linear retention index; RT, retention time.

Similar to acetic acid, the content of furans and furanic compounds in coffee beans was significantly improved by roasting degree. Carbohydrates and amino acids are two typical precursors with a relatively high concentration in green coffee beans (Chaichi et al., 2015). During roasting, furans, such as 2‐furanmentaol (furfuryl alcohol), are yielded from the reaction between sucrose, ribose, or deoxyosones and amino acids (cysteine or methionine), which could be partially responsible for the caramel aroma of roasted coffee beans (Caporaso et al., 2018; Hertz‐Schunemann et al., 2013; Sanz et al., 2002; Somporn et al., 2011). Furanic compounds including furfural and 5‐methylfurfural could also contribute to coffee aroma and come from two pathways (Chaichi et al., 2015). One is derived from the dehydration, cyclization, and polymerization of Amadori rearrangement products after the Maillard reaction, especially deoxyribose (Caporaso et al., 2018). Furanic compounds could also be obtained from the thermal oxidation of furfuryl alcohol, polyunsaturated fatty acids, and ascorbic acid (Anese, 2015; Caporaso et al., 2018; Chaichi et al., 2015). Therefore, the content of furfural and 5‐methylfurfral could be promoted dramatically by the improvement of furfuryl alcohol formation, when the roasting degree is enhanced from light to dark, which fits the results of this research.

A clustering was found in the group of pyrazines according to Table 3 because 2,5‐dimethylpyrazine and 2,6‐dimethylpyrazine generate from the same Maillard reaction with different locations of the functional groups (Baggenstoss et al., 2008; Caporaso et al., 2018; Lee et al., 2016). The content of pyrazines was observed generally stable among three roasting degrees except for some fluctuations. Normally, the content of pyrazines reaches a peak when the temperature is around 250°C. Some other researchers assumed that pyrazines would be incorporated into melanoidins while the temperature is above 250°C, which would lead to a reduction in the pyrazines content (Schenker et al., 2002).

Pyrroles and pyridines, typical roasting products identified in this research, showed an increased tendency related to increasing roasting degree. The principle of these two group compounds formation in roasted coffee beans is similar as that of pyrazines. The Strecker reaction between aldoses (aldehydes) and alkylamines (aminoketones) would occur subsequently when other amino acids take part in, followed by heterocyclization and generating a series of aroma active volatile compounds including pyrroles, pyridines, and pyrazines (Caporaso et al., 2018; Hertz‐Schunemann et al., 2013). Pyridine could also be derived from the degradation of trigonelline (Baggenstoss et al., 2008; Hertz‐Schunemann et al., 2013). Therefore, the content of pyridine would be reduced by overroasting.

The tendency of phenol content increased with an intensive roasting degree as well in this research. This is probably because of the formation of phenol in roasted coffee beans which is through the degradation of caffeoylquinic acid and ferulic acid originated from the decomposition of chlorogenic acids when roasting is exothermic (Baggenstoss et al., 2008; Caporaso et al., 2018). Generally, suitable roasting with high intensity could improve the content of volatile compounds in coffee beans which is beneficial to the development of coffee beans flavor.

### 3.4 Antioxidant activities of roasted coffee beans estimation (DPPH, FRAP, ABTS, ^·^OH‐RSA, FICA, and RPA)

The results of antioxidant activity (DPPH, FRAP, ABTS, ^·^OH‐RSA, FICA, and RPA) for the estimation of antioxidant capacity of coffee beans were analyzed as shown in Table 1. Overall, all six assays result of coffee beans with increasing roasting degree showed basically no significant differences statistically except some fluctuations. It agrees with previous research that antioxidant activity would not linearly increase with increasing roasting temperature (Odzakovic et al., 2016; Somporn et al., 2011).

DPPH and ABTS primarily have been used for determining antioxidant activity, especially free radical scavenging capacity via hydrogen atom transferring (Du et al., 2021; Górnaś et al., 2015; Nebesny & Budryn, 2003; Sirivibulkovit et al., 2018). For free phenolic compounds, coffee beans with increasing roasting degrees exhibited similar DPPH values around 145 mg TE/g with no significant difference. Interestingly, the bound DPPH value of light‐roasted coffee beans was the lowest with 69.98 ± 2.26 mg TE/g, whereas dark roasted showed the highest with 77.39 ± 0.89 mg TE/g. Combined with the changes in the estimation of their phenolic content, the free radical scavenging capacity probably depends more on the flavonoids owing to their structure, such as the 3’,4’‐dihydroxy system of the B‐ring in quercetin (Choi et al., 2002). Regarding previous studies, some novel substances with outstanding antioxidant activities could be generated by Maillard reaction during roasting, such as melanoidins (del Castillo et al., 2002; Odzakovic et al., 2016). This would be the main reason for the maintenance and improvement of antioxidant activities. The values of free DPPH were more than two times that of the bound which is probably because of the releasement of bound phenolic compounds during roasting.

No significant differences but some fluctuations were observed among the free ABTS values of different roasted coffee beans along with the increasing roasting temperature. Medium‐roasted coffee beans performed the highest free ABTS value with 102.37 ± 8.10 mg TE/g, followed by the light and dark roasted. A similar trend was shown in the bound ABTS results, whereas the light‐roasted coffee beans got the lowest value with 61.51 ± 2.20 mg TE/g. The results agreed with previous research done by del Castillo et al. (2002), van der Werf et al. (2014), and Odzakovic et al. (2016) that medium‐roasted coffee beans obtained the highest free and bound ABTS value. With the combination of Figure 1 and MS/MS results, it was indicated again that novel compounds with antioxidant activity could be generated when the degradation of some phenolic compounds occurs during the slightly intensive roasting.

RPA and FRAP assays could be both used to estimate the reducing capacity of antioxidants via reducing Fe^3+^ into Fe^2+^ by electron transfer (Ali et al., 2021; Subbiah et al., 2020). For free phenolic compounds, although the FRAP results of the dark‐roasted coffee beans (29.50 ± 0.52 mg TE/g) performed slightly lower than that of the light roasted (30.34 ± 1.03 mg TE/g), there was no significant difference between them. The reducing capacity of dark‐roasted coffee beans achieved the highest in RPA assay at 52.18 ± 1.38 mg TE/g after a slight reduction in that of the medium roasted. However, there was no significant difference in the comparison between light‐ and medium‐roasted coffee beans statistically. For bound phenolic compounds extract, the values of both assays increased firstly at medium roasting and then slightly declined at dark roasting with no significant difference. It is partially different from Pokorná et al. (2015), who concluded that FRAP would gradually decrease by continuing roasting. However, it is consistent with previous research conducted by Herawati et al. (2019), which indicate that the reducing capacity could come from the native and novel‐formed bioactive compounds.

Different from the previous four assays, ^·^OH‐RSA assay is based on Fenton and Haber–Weiss reaction, in which ferrous ion (Fe^2+^) would react with hydrogen peroxide and generate ferric ion (Fe^3+^) and hydroxyl radical which would be scavenged by antioxidants (Chou et al., 2021). The tendency of this assay results was increased when the roasting degree was enhanced. Both free and bound phenolic compounds in the dark‐roasted coffee beans performed the highest capacity of hydroxyl radical scavenging around 32.94 ± 0.29 and 52.11 ± 2.07 mg TE/g. Similar result was obtained by Budryn et al. (2017), which is roasted coffee extract under 230°C was the most active in OH radicals scavenging. Budryn et al. (2017) also concluded that the high antioxidant activity of roasted coffee beans could also be determined by other compounds, such as hydrophilic Maillard reaction products, not only by chlorogenic acids.

During FICA assays, the antioxidants interfere with the formation of ferrous and ferrozine complex by chelating ferrous ions and result in the drop off of the color complex. Hence, the reduction in the color intensity could be in equivalence to its metal chelating activity (Patel, 2013; Santos et al., 2017). In this study, the bound FICA values of all three roasted coffee beans were similar, which were basically maintained along with intensive roasting. However, from light‐ to dark‐roasted degree, the free FICA values slightly increased from 0.37 ± 0.09 to 0.51 ± 0.03 mg EE/g but exhibiting no significant difference statistically.

Generally, the antioxidant activity of coffee beans would decrease along with intensive thermal processing owing to the degradation of polyphenolic compounds (Hecimovic et al., 2011). However, suitable thermal processing could change the structure of existed antioxidants or catalyze the formation of novel antioxidant compounds so that maintain or enhance the antioxidant capacity (Cho et al., 2014; Somporn et al., 2011). For instance, melanoidins, reductive ketones, and other heterocyclic compounds, which are displayed as effective antioxidants, could be formed through Maillard reaction during roasting (Cho et al., 2014; Delgado‐Andrade & Morales, 2005). Moreover, phenylindans and other polyphenol derivatives with high antioxidant capacity could be generated (Hecimovic et al., 2011). Although lower molecular mass polyphenols were generated via the degradation of high molecular mass polyphenols, such as phenolic acid, improve the overall antioxidant capacity. The metal chelation activity of these high molecular mass polyphenols would be considerably reduced, which is reflected by FICA assays (Cho et al., 2014; Kim et al., 2011).

### 3.5 Correlation between phenolic compounds and antioxidant potential

Pairwise Pearson's correlation test was performed to evaluate whether the content of phenolic compounds in coffee beans contributed to their related antioxidant activities. The correlation test results were shown in Table 4. TPC was significantly positively correlated with most antioxidant potential estimation assays that the absolute values of the *r* values for the pairwise correlations were higher than 0.7. With the consideration of a small sample size, the absolute value of the correlation coefficient closer to 1 represents the stronger tendency (Sedgwick, 2012). Therefore, it indicated that phenolic compounds within the coffee beans extract may be the primary constituents responsible for the antioxidant capacity of coffee beans (Wang et al., 2009).

**TABLE 4:** Pearson's correlation coefficients (*r*) of phenolic contents and the antioxidant capacity

Variables	TPC	TFC	TCT	DPPH	FRAP	ABTS	^·^OH‐RSA	FICA
TFC	0.467							
TCT	−0.193	0.135						
DPPH	0.786^a^	0.601^b^	−0.524					
FRAP	0.785^a^	0.683^b^	−0.449	0.936^a^				
ABTS	0.747^a^	0.486	−0.525	0.954^a^	0.916^a^			
^·^OH‐RSA	−0.932^a^	−0.535	0.221	−0.847^a^	−0.844^a^	−0.840^a^		
FICA	−0.781^a^	−0.633^b^	0.523	−0.990^a^	−0.975^a^	−0.941^a^	0.859^a^	
RPA	0.704^b^	0.637^b^	−0.517	0.964^a^	0.953^a^	0.908^a^	−0.742^a^	−0.969^a^

^a^ Significant correlation with *p* ≤ .01.

^b^ Significant correlation with *p* ≤ .05.

TFC assay performed a significant positive correlation with DPPH, FRAP, and RPA, and negative with FICA. It agreed with previous research conducted by Amin et al. (2013). They stated that the reducing characteristics are commonly related to the presence of reductones which could donate a hydrogen atom and then break the free radical chain to conduct antioxidant action.

Interestingly, TCT exhibited a moderate, negative correlation with other antioxidant assays except for TFC, ^·^OH‐RSA, and FICA assays. It could be inferred that roasting could degrade condensed tannins into the lower molecular mass of flavonoids and slightly improve the TFC values, which is consistent with the previous conjecture. Moreover, it seemed like low degradation of condensed tannins was not enough to maintain the antioxidant capacity with increased roasting temperature.

All assays for the estimation of antioxidant potential showed a significant positive correlation with each other, whereas performed significant negative correlation with ^·^OH‐RSA and FICA. The antioxidant in both assays plays a role as the chelating agent. Hence, the hydroxyl radicals scavenging capacity of coffee beans was probably determined more on the novel antioxidant compounds. The metal chelating activity of the high molecular mass of phenolic compounds could be crippled after thermal degradation while still could relieve oxidation via other pathways (Cho et al., 2014).

## 4 CONCLUSION

According to the current study, it was found that commercial light‐roasted coffee beans displayed a relatively higher content of total phenolic compounds (TPC) and antioxidant potential (DPPH, ABTS, FRAP, and FICA). The dark‐roasted exhibited higher content of total flavonoids and condensed tannins (TFC and TCT), as well as the better capacity of scavenging hydroxyl radical and reducing power (^·^OH‐RSA and RPA). Nevertheless, the commercial medium‐roasted coffee beans were overall better in all estimation of phenolics content and antioxidant potential. From the advanced LC‐ESI‐QTOF‐MS/MS analytical technique applied for the identification and characterization of the phenolic compounds in roasted coffee beans, a total of 23 phenolic compounds were tentatively identified in our study. Most phenolic compounds were detected in the medium‐roasted coffee beans. As for the GC‐MS, a total of 20 volatile compounds were identified and quantified in all roasted coffee beans. Generally, the dark‐roasted coffee beans performed the highest value of all detected volatile compounds, followed by the medium roasted closely. In conclusion, the content of phenolic compounds in coffee beans would decline along with the intensification of roasting. The antioxidant activities of coffee beans could be at least maintained or improved to some extent even after intensive roasting owing to the generation of novel substances with outstanding antioxidant activity. The medium‐roasted coffee beans contain the most various phenolic compounds and relatively outstanding aroma properties.

## CONFLICT OF INTEREST

The authors declare no conflict of interest.

## Supporting information

Supplementary Material

## Data Availability

Data available in article supplementary material.
