# Supplementary material for: Impact of roasting on the phenolic and volatile compounds in coffee beans
Source: Food Sci Nutr. 2022 Apr 1;10(7):2408–25. doi: 10.1002/fsn3.2849 (PMC9281936; doi:10.1002/fsn3.2849)
Supplement: Supplementary file 1 — Supplementary Material [file FSN3-10-2408-s001.docx]

*Supplementary data*

**Impact of roasting on the phenolic and volatile compounds in coffee beans**

**ABSTRACT**

Phenolic compounds presenting in coffee beans could generate flavor and bring benefits to health. This study aimed to evaluate the impacts of commercial roasting levels (light, medium and dark) on phenolic content and antioxidant potential of Arabica coffee beans (*Coffea arabica*) comprehensively via antioxidant assays. The phenolic compounds in roasted samples were characterized via liquid chromatography-electrospray ionization quadrupole time-off light mass spectrometry (LC-ESI-QTOF-MS/MS). Further, the coffee volatile compounds were identified and semi-quantified by headspace/gas chromatography-mass spectrometry (HS-SPME-GC-MS). Generally, for phenolic and antioxidant potential estimation, light roasted samples exhibit the highest TPC (free: 23.97 ± 0.60 mg GAE/g; bound: 19.32 ± 1.29 mg GAE/g), DPPH and FRAP. The medium roasted beans exhibited the second high in all assays but the highest ABTS^+^ radicals scavenging capacity (free: 102.37 ± 8.10 mg TE/g; bound: 69.51 ± 4.20 mg TE/g). Totally, 23 phenolic compounds were tentatively characterized through LC-ESI-QTOF-MS/MS which is mainly adopted by 15 phenolic acid and 5 other polyphenols. Majority of phenolic compounds were detected in the medium roasted samples, followed by the light. Regarding GC-MS, a total of 20 volatile compounds were identified and semi-quantified which exhibited the highest in the dark followed by the medium. Overall, this study confirmed that phenolic compounds in coffee beans would be reduced with intensive roasting whereas their antioxidant capacity could be maintained or improved. Commercial medium roasted coffee beans exhibit relatively better nutritional value and organoleptic properties. Our results could narrow down previous conflicts and be practical evidence for coffee manufacturing in food industries.

**KEYWORDS:** *Coffea arabica*, roasting, phenolic compounds, antioxidant properties, volatile compounds, characterization, semi-quantification, LC-MS/MS, GC-MS

**(a)**

**(b)**

**(c)**

**(d)**

**(e)**

**(f)**

**Figure 1S:** LC-ESI-QTOF-MS/MS basic peak chromatograph (BPC) for characterization of phenolic compounds of roasted coffee beans; **(a)** Light roasted coffee beans in negative ionization mode; **(b)** Medium roasted coffee beans in negative ionization mode; **(c)** Dark roasted coffee beans in negative ionization mode; **(d)** Light roasted coffee beans in positive ionization mode; **(e)** Medium roasted coffee beans in positive ionization mode; **(f)** Dark roasted coffee beans in positive ionization mode.

**(A)**

**(B)**

**Figure 2S.** The LC-ESI-QTOF-MS/MS characterization of 2,3-dihydroxybenzoic acid; (**A**) A chromatograph of 2,3-dihydroxybenzoic acid (Compound 1, RT: 14.815, Table 2), in the negative mode of ionization [M – H]^−^ identified in DRL; (**B**) Mass spectra of 2,3-dihydroxybenzoic acid with observed/precursor of *m/z* 153.0198.

**(A)**

**(B)**

**Figure 3S.** The LC-ESI-QTOF-MS/MS characterization of 2-hydroxybenzoic acid; (**A**) A chromatograph of 2-hydroxybenzoic acid (Compound 2, RT: 18.259, Table 2), in the negative mode of ionization [M – H]^−^ identified in DRL; (**B**) Mass spectra of 2-hydroxybenzoic acid with observed/precursor of *m/z* 137.0249.
